# About Appointment Books

**Published:** 1882-01

**Authors:** 


### EDITORIAL, ETC.

*About Appointment Books*—W. A. Dartt, D. D. S., of Milwaukee, Wisconsin, has made a very complete appointment book, the using of which will, the writer trusts, save him some vexation and money also. It is not an infrequent thing for fillings to be inserted, and in the haste or forgetfulness of the moment, one or more are forgotten and so remain unchanged. Dr. Dartt's book provides a place for the names of ten patients a day, quite as many as the most industrious dentist will be likely to work for, or indeed, ought to work for, at hours from 8 A. M., to 5 P. M. Columns for gold, amalgam, os-artificiel, gutta-percha and tin fillings, respectively, are in one division of the page; and a place for anæsthetics, extraction. treatment, crowns, cleanlng, artificial teeth, number of hours devoted to each, with debit, credit and expense accounts. It embraces, in a word, everything save registration of the location of fillings, and has a page for every day in the year except Sunday, with dates left blank, so that the book may begin at any time. In this way one may enter opposite the patient's name the work done, or if desirable to use the book as such, work *to be* done, (in pencil) which may be written out afterwards in ink. We feel that this is to be a helpful book, and we shall begin the new year with its use. There is a Dental Ledger also published by Dr. Dartt. H.

